# PECUU-ECM Patches: The Future of Ischemic Heart Disease Repair∗

**DOI:** 10.1016/j.jacbts.2021.03.003

**Published:** 2021-05-24

**Authors:** Yasmeen Ajaj, Ngozi Damilola Akingbesote, Yibing Qyang

**Affiliations:** a Biological and Biomedical Sciences Program, Yale University, New Haven, Connecticut, USA; b Department of Internal Medicine, Section of Cardiovascular Medicine, Yale Cardiovascular Research Center, Yale School of Medicine, New Haven, Connecticut, USA; c Yale Stem Cell Center, New Haven, Connecticut, USA; d Vascular Biology and Therapeutics Program, Yale University School of Medicine, New Haven, Connecticut, USA; e Department of Pathology, Yale School of Medicine, New Haven, Connecticut, USA

**Keywords:** biomaterial, cardiac patch, left ventricular remodeling, myocardial infarction

Myocardial infarction leads to pathological changes in the heart, including loss of cardiomyocytes and degradation of the cardiac extracellular matrix (ECM). These changes result in wall thinning, infarct enlargement, and development of scar tissue. Such remodeling ultimately leads to end-stage heart failure (1,2). To address this problem, researchers have implemented the use of biomaterials such as decellularized ECM, hydrogels, and scaffolds to create surrogate sources for replacing damaged tissue in ischemic heart disease (1,2). These biomaterials have been implemented through two approaches. The first approach involves the production of cardiac patches in vitro that are applied to the diseased area. The second approach involves the use of injectable biomaterials that halt negative cardiac remodeling and recruit endogenous cells to the diseased tissue for its repair (2). While both approaches have shown promise in cardiac tissue engineering, there are still limitations to each. For example, although many cardiac patches have the structural scaffold to provide mechanical support to diseased tissue, they lack the robust ability to recruit endogenous cell types to the damaged area (1,2). On the other hand, injectable materials such as decellularized matrix and hydrogels that are capable of recruiting endogenous cell types for repair may be too stiff or too weak to produce the required relief (1,2).

In order to address these limitations, Silveira-Filho et al. (3) investigated the efficacy of biohybrid patches made from the degradable elastomer poly(ester carbonate urethane)urea (PECUU) and an ECM-based hydrogel devoid of live cell components (3). These PECUU-ECM biohybrid patches combine the advantages of a biodegradable polymer scaffold that provides higher anisotropy and mechanical strength with the advantages of a decellularized ECM composed of bioactive growth factors and matricellular proteins that enable bioinductive effects in the diseased tissue (3). Their study compared the outcomes of 3 groups of rats: non-patched infarcted rats; infarcted rats surgically administered a PECUU-ECM patch implant; and non-patched infarcted rats orally administered losartan, an AT1R blocker used as standard therapy in ischemic heart disease (3). The left ventricles (LVs) of infarcted rats with PECUU-ECM patch implants more closely resembled healthy LVs and were less spherical at 8 weeks post-implant than both of the nonpatched groups (3). These patched animals showed improvements in ejection fraction and percentage of fractional shortening from pre-treatment to 4 and 8 weeks post-treatment compared to the nonpatched groups. Notably, a subgroup of the sickest rats with the lowest post-infarct ejection fraction also showed improvements after 8 weeks of treatment with the PECUU-ECM patches (3). Histological sections of infarcted LVs with PECUU-ECM patch implants were observed to have considerable cellular infiltration, a feature that was absent in the control infarcted groups (3). Surprisingly, LV scar areas did not show any differences based on treatment. However, it was observed that the infarct to healthy tissue size ratio was lower in PECUU-ECM-treated animals than in the control groups (3). Silveira-Filho et al. (3) convincingly showed that the PECUU-ECM patches have the ability to improve cardiac function in chronic ischemic heart disease with extensive post-infarction remodeling.

Many studies in the field of cardiac tissue engineering address remodeling timepoints immediately after ischemic wounding. However, this acute approach is not realistic because most of the remodeling in clinical ischemic heart disease occurs undetected over long periods of time, leading to extensive ventricular thinning, scarring, and progressive heart failure. The experimental time course followed in this study attempted to recapitulate extensive remodeling in the validation of PECUU-ECM patches by addressing ischemic wounds 8 weeks after myocardial infarction. This study found PECUU-ECM patches were efficacious in reversing the negative remodeling seen in advanced stages of ischemia, offering great potential for clinical translation. Additionally, the authors’ rationale for incorporating PECUU patches within the biohybrid patch was its slower degradation rate relative to that of poly(ester urethane)urea (PEUU). This slower degradation rate ultimately led to better remodeling outcomes in post-infarcted rats given a PECUU patch compared to those given a PEUU patch over their infarcted hearts. Despite the slower rate of degradation, PECUU patches favorably did not form encapsulations typically caused by nondegradable material. These findings point to the importance of polymer selection in the development of cardiac patches. Notably, there are still some challenges this study needs to address in the future. The biohybrid patch used is mechanically compatible with rat myocardium, yet how these patches may influence the contractility and electrophysiology of human myocardium and whether the patches will withstand the pressures of a full adult human cardiac load upon implantation remain to be investigated (2). If implemented in human patients, these patches would have to be thicker to withstand mechanical demands, which may affect the diffusion of oxygen and nutrients into the myocardium due to the lack of pre-established vasculature (2). As suggested by the authors, implementing the PECUU-ECM biohybrid patches in a larger animal model is critical to addressing these outstanding questions and optimizing this technology for future clinical use. Another important consideration is that porcine cardiac ECM components used in the construction of these biohybrid patches differ in composition and kinetics compared to rat ECM. Thus, the ventricular remodeling arising from native rat host cell infiltration into a porcine ECM may not be optimized for these rat studies. It would be of interest to investigate the remodeling outcomes of a biohybrid patch synthesized with ECM derived from the same or a similar species in which it is implanted.

Recent advances in the field of cardiac tissue engineering include the use of human induced pluripotent stem cell (iPSC)-derived cardiomyocytes to produce engineered heart tissue (EHT) (4). In the future, these optimized EHTs could then be decellularized and the remaining ECM used for human-derived PECUU-ECM biohybrid patch synthesis optimized for the remodeling and mechanical demands of the human heart. This approach may reduce the batch-to-batch variability that arises from using porcine- or human cadaver-derived ECM, as well as ensure the use of healthy ECM components. The ventricular remodeling outcomes in this study result from the repopulation of the healthy native host cells in the implanted patch. Notably, in clinical settings, myocardial infarctions are more prevalent in patients who are elderly, have reduced myocardial regenerative potential, or have pre-existing diseases. These conditions exclude such patients from being candidates for cell-free PECUU-ECM patch implants. One putative solution to this problem is the use of nonimmunogenic iPSCs with altered human leukocyte antigen expression (5). These “universal” iPSCs may be used to derive healthy regenerative cardiomyocytes that could be seeded onto PECUU-ECM patches and undergo short-term culture prior to implantation on infarcted myocardium. Large-scale production of these allogeneic human iPSC (hiPSC)-derived PECUU-ECM patches would allow for immediate patient treatment upon infarction diagnosis with limited complications arising from host rejection. In addition, these hiPSC-derived biohybrid patches could be further optimized for human implantation by using ECM derived from human EHT.

The PECUU-ECM technology engineered by Silveira-Filho et al. (3) shows promise for use in clinical treatment of chronic post-infarction cardiac remodeling. Their paper lays the groundwork for future investigations into efficacious PECUU-ECM patch parameters in larger model organisms. Ultimately, this work could potentially contribute to the large-scale development of immediately available off-the-shelf products that could be used for patient care in the future.

## Funding Support and Author Disclosures

The authors have reported that they have no relationships relevant to the contents of this paper to disclose.
